# Contribution of viral infection to risk for cancer in systemic lupus erythematosus and multiple sclerosis

**DOI:** 10.1371/journal.pone.0243150

**Published:** 2021-01-22

**Authors:** Deborah K. Johnson, Kaylia M. Reynolds, Brian D. Poole, Matthew D. Montierth, Vera M. Todd, April Barnado, Mary F. Davis

**Affiliations:** 1 Department of Microbiology and Molecular Biology, Brigham Young University, Provo, UT, United States of America; 2 Division of Rheumatology & Immunology, Vanderbilt University Medical Center, Nashville, TN, United States of America; 3 Department of Biomedical Informatics, Vanderbilt University Medical Center, Nashville, TN, United States of America; Istituto Nazionale Tumori IRCCS Fondazione Pascale, ITALY

## Abstract

Patients with autoimmune disorders (AD) have altered cancer risks compared to the general population. Systemic lupus erythematosus and multiple sclerosis lead to a heightened risk for hematological malignancies and decreased risk for breast, ovarian, and prostate malignancies. Often patients with autoimmune disease have dysregulated antiviral immune responses, including against oncogenic viruses. To uncover the relationship between viral incidence and cancer risk in the context of autoimmune disease, we extracted electronic health records (EHR) from Vanderbilt University. ICD-9/10 codes and laboratory values were collected for hematological, lung, anal-vaginal, thyroid, hepatobiliary, bladder, prostate, and breast cancers; and viruses including Epstein Barr virus (EBV), Human papilloma virus (HPV), and Hepatitis A/B/C (Hep). Only viral infections that led to a physician visit or laboratory test were entered into the EMR; therefore, only clinically relevant cases were noted and considered positive in this study. The relationship between virus infection and cancer in an SLE cohort (SLE-cases n = 2,313, and SLE-controls n = 5,702) and an MS cohort (MS-case n = 7,277, MS-control n = 7,277) was examined by multilinear logistic regression. Viral infection was strongly associated with increased risk for cancer overall. SLE and MS patients were more susceptible to all viral infections. MS patients trended toward increased risk for cancers overall, while decreased risk for hormone-based cancers in SLE patients non-significantly reduced their risk for overall cancer. Both SLE and MS patients had increased clinically relevant EBV infection, which was associated with risk for hematological cancers. Preventing viral infections by vaccination may be especially helpful in controlling risk for cancer in SLE and MS patients.

## Introduction

Systemic lupus erythematosus (SLE) is a common, debilitating, and complex systemic autoimmune disease (AD) primarily affecting women of childbearing age. Its diverse symptoms include arthritis, fatigue, rash, sensitivity to sunlight, and in severe cases, kidney damage, blood disorders, neurological damage, and death. While SLE survival has improved, disease- and treatment-related mortality and morbidity remain substantial. SLE patients have an increased risk for certain cancers [1] despite having a lower risk for hormonally-based cancers such as breast, ovarian and prostate cancers [2,3]. SLE patients may also be more susceptible to viral infections, including viruses associated with cancer such as Epstein-Barr virus or Human Papilloma virus [4,5].

It is unclear how the overall cancer risk of SLE patients is affected by the relative contributions of SLE itself, its treatments, and other environmental factors such as increased viral infection [6,7]. Compared to the general population, SLE patients experience increased incidence of hematological, hepatobiliary, vulvar, vaginal, and cervical cancers, all of which have conspicuous links to viruses [8,9]. SLE patients may lack viral control due to immune dysfunction. For example, SLE T cells are dysregulated in response to Epstein-Barr virus (EBV) [10,11]. Similar dysregulated immune response to EBV contributes to Burkitt’s lymphoma [12]. EBV infection is also associated with non-Hodgkin’s lymphoma and diffuse large B cell lymphoma [13,14]. The increased EBV viral loads in SLE patients [15] may help explain the increased incidence of hematological cancers.

EBV is also implicated in the development of multiple sclerosis (MS) [16], a chronic autoimmune inflammatory disease affecting the central nervous system that, like SLE, primarily affects women of childbearing ages [17]. Furthermore, similar to SLE, immunosuppressive treatment in MS may alter the risk of viral infection and cancer development compared to the general public [18,19]. While MS patients have a decreased risk for ovarian and prostate cancers, hematological cancers are consistently increased, again suggesting that poor viral control may lead to cancer development [20].

This study seeks to understand the contributions of viral infections to cancer development in SLE and MS patients. The risk of viral infections and cancers were compared by logistic regression using electronic health records examining ICD-9 and -10 billing codes and laboratory values from Vanderbilt University Medical Center’s electronic health record database. Most people with a viral infection will not be tested for that infection, and the vast majority of people are positive for EBV infection. Therefore, this study uses positive viral tests as a proxy for severe or clinically relevant viral infection rather than simple infection, which would be expected in most people. Our results demonstrate that viral status within an autoimmune population more fully predicts cancer risk than autoimmunity alone.

## Materials and methods

### Study population

**Fig 1:**
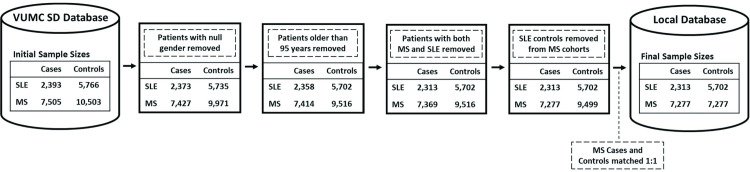
Filtering procedure for MS and SLE cohorts.

Patient demographic information, virus laboratory results, and ICD-9/10 billing codes were extracted from Vanderbilt University Medical Center Synthetic Derivative (VUMC SD) database, which contains de-identified electronic health records for over 3 million patients seen at Vanderbilt University Medical Center. The study was approved by the Vanderbilt University Institutional review board. We used a previously identified systemic lupus erythematosus (SLE) cohort with matched controls by age, race and gender (Fig 1) [21]. These SLE cases were identified using previously validated and published algorithms with positive predictive values (PPVs) ≥ 90% [22]. Controls were subjects who had ≥ 3 outpatient visits in the past 5 years at VUMC. Controls were frequency matched based on age, race, and gender. Multiple sclerosis (MS) cases were identified with a computer algorithm as previously described [23]. Briefly, selected MS control patients did not have any ICD codes for other ADs and were matched by age in 2018, race, and gender (Table 1). Age, race, and sex matched controls were chosen from the Synthetic Derivative database for each MS patient. Patients missing values for their sex, who were older than 95 years in 2018, or who had both SLE and MS were excluded. Furthermore, MS case and control patients were removed if they overlapped with the SLE controls. The final MS study cohort consisted of 7,277 cases and 7,277 controls, and the SLE final study groups was composed of 2,313 cases and 5,702 controls (Fig 1).

**Table 1:** Population characteristics.

		*SLE Case*	*SLE Control*	*MS Case*	*MS Control*
	*Total (n)*	2313	5702	7277	7277
**Demographic Information**	*Female*	2058 (89%)	5132 (90%)	5603 (77%)	5167 (71%)
	*Age*	55.0 ± 16.8	52.3 ± 16.6	56.7 ± 12.8	54.4 ± 17.8
	*Asian*	36 (2%)	123 (2%)	20 (0%)	88 (1%)
	*African American*	535 (23%)	1419 (25%)	659 (9%)	606 (8%)
	*Hispanic*	57 (2%)	166 (3%)	66 (1%)	111 (2%)
	*Native American*	2 (0%)	5 (0%)	6 (0%)	2 (0%)
	*Caucasian*	1552 (67%)	3729 (65%)	5461 (75%)	4503 (62%)
	*Other*	6 (0%)	16 (0%)	7 (0%)	43 (1%)
	*2+ Ethnicities*	5 (0%)	7 (0%)	8 (0%)	6 (0%)
**Viral Status**	*Unknown*	120 (5%)	237 (4%)	1050 (14%)	1918 (26%)
	*None*	2016 (87%)	5194 (91%)	7116 (98%)	7221 (99%)
	*Any*	297 (13%)	508 (9%)	161 (2%)	56 (0.8%)
	*Hep A/B/C +*	198 (9%)	326 (6%)	20 (0.3%)	9 (0.1%)
	*EBV+*	163 (7%)	196 (3%)	115 (2%)	33 (0.5%)
	*HPV+*	30 (1%)	62 (1%)	28 (0.4%)	15 (0.2%)
**Cancer Status**	*All*	232 (10%)	571 (10%)	297 (4%)	236 (3%)
	*Breast*	39 (2%)	175 (3%)	89 (1%)	68 (0.9%)
	*Prostate*	8 (0.3%)	48 (0.8%)	32 (0.4%)	37 (0.5%)
	*Hematological*	163 (7%)	203 (4%)	124 (2%)	65 (0.9%)
	*Lung*	17 (0.7%)	63 (1%)	44 (0.6%)	36 (0.5%)
	*Thyroid*	12 (0.5%)	52 (0.9%)	24 (0.3%)	12 (0.2%)
	*Anal/Vagina/Cervical*	10 (0.4%)	46 (0.8%)	11 (0.2%)	13 (0.2%)
	*Hepatobiliary*	4 (0.2%)	31 (0.5%)	3 (0.04%)	9 (0.1%)
	*Bladder*	3 (0.1%)	24 (0.4%)	7 (0.1%)	8 (0.1%)

Demographic information, viral incidence and cancer incidence for SLE and MS cohorts by case and control.

### Identifying malignancies and viral infections

Patients were classified as having a malignancy (hematological, lung, vaginal, anal, hepatobiliary, bladder, thyroid, breast, or prostate) if their records contained at least two ICD-9/10 billing codes for a specific cancer (Table 1). Anal and vaginal cancers are both associated with HPV infection, and thus were grouped to achieve sufficient patient numbers for analysis. To confirm viral infection history, patients were assigned positive viral status if they had a minimum of two instances of a positive laboratory value (serological or PCR based) and/or ICD-9/10 billing code in their records (Table 2). More viral infections were detected in the SLE cohort than the MS cohort (Table 1). Hepatitis (Hep) includes both hepatitis B and hepatitis C infections.

**Table 2:** ICD-9 /10 and lab codes.

**Cancer Codes**	**ICD-10**	**ICD-9**
*Hematological*	C81, C82, C83, C84, C85, C86, C87, C88, C89, C90, C91, C92, C93, C94, C95, C96, D46, D47, Z85.79	200–207 by 0.01, 273, 287.30, 287.3
*Lung*	C34, Z85.11	162–163 by 0.01
*Anal/Vaginal/Cervical*	C51, C52, C53, C21	180–181 by 0.01, 184.0, 184.4, 154.2, 154.3, 154.8
*Hepatobiliary*	C22, C23, Z85.05	155–157 by 0.01
*Bladder*	C67, Z85.51	188–189 by 0.01
*Thyroid*	C73, Z85.85	193–194 by 0.01
*Breast*	C50, Z85.3	174–176 by 0.01
*Prostate*	C61, D07.5, Z85.46	185–186 by 0.01
**Viral Codes**	**ICD-10**	**ICD-9**
*EBV*	B27	75–76 by 0.01
*HPV*	B97.7, B85.81, B85.82, R87.81, R87.82, R85.81, R85.82	079.4, 78.11, 795.05, 795.15, 796.75, 795.79, 795.099, 795.19
*Hep*	B15, B16, B17, B18, B19	70–71 by 0.01
**Lab Viral Names**		
*EBV*	Epstein-Barr Virus	
*HPV*	Human Papilloma Virus	
*Hep*	Hepatitis C Virus, Hepatitis B Virus	

ICD-9 /10 and lab codes used to identify patient cancer and viral incidence.

### Statistical analysis

Multilinear logistic regression models were used to predict overall viral incidence and cancer risk, and calculate risk for individual viruses and cancers (Table 3). To account for multiple testing, p-values were adjusted via a Bonferroni correction by multiplying the p-value by the number of regressions run for each autoimmunity group (SLE = 8, MS = 4). All filtering and statistical tests were completed in R 3.6.2.

**Table 3:** Logistic regression models.

Response Variable	Disease Status	Gender	Age	Viral Infections	Interaction
*Viral Status*	SLE/MS	M/F	Age	None	None
*Malignancy Status*	SLE/MS	M/F	Age	EBV, HPV, HEP	(SLE/MS)*Viral Status
*Hematological Status*	SLE/MS	M/F	Age	EBV	(SLE/MS)*EBV
*Lung Status*	SLE	M/F	Age	EBV, HPV, HEP	SLE*Viral Status
*Anal/Vaginal Status*	SLE	F	Age	HPV	SLE*HPV
*Breast Status*	SLE	F	Age	EBV, HPV, HEP	SLE*Viral Status
*Prostate Status*	SLE	M	Age	EBV, HPV, HEP	SLE*Viral Status

Covariate and cohort specifications for each regression performed.

## Results

### Overall viral incidence and cancer incidence

**Fig 2:**
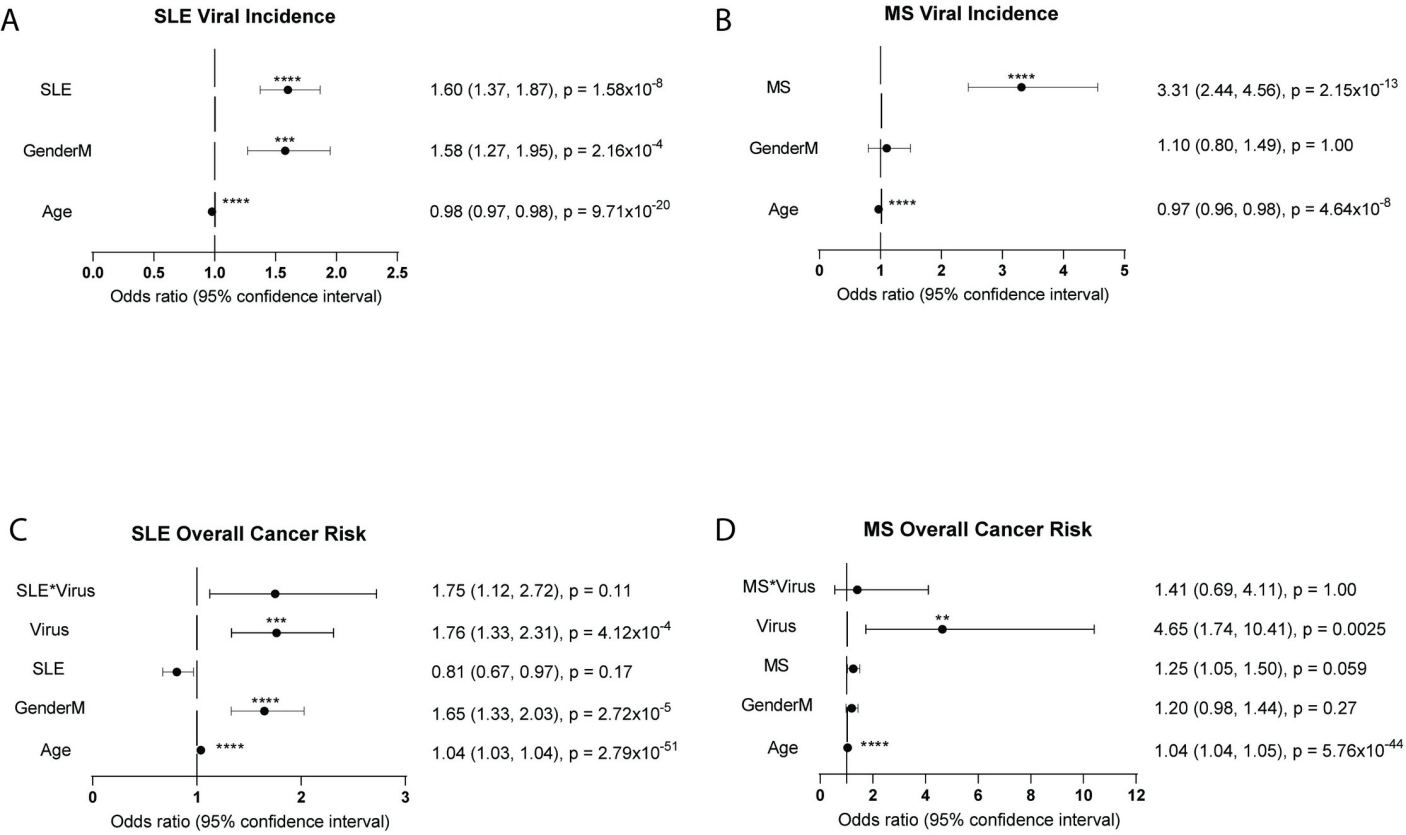
Autoimmune disorders increase viral incidence. Viral incidence increases overall cancer risk within the autoimmune cohorts. Odds ratios (OR) and 95% confidence intervals of SLE and MS cohorts. For all graphs, the dotted line represents an OR of 1.0. An OR to the right of the dotted line represents an increase in risk. An OR to the left of the solid line represents a reduction in risk. (a) Overall viral incidence is significantly predicted by SLE (OR 1.60, 95% CI 1.37, 1.87, p = 1.58x10^-6^) and being male with SLE (OR 1.58, 95% CI 1.27, 1.95, p = 2.16x10^-4^). (b) MS predicts increased positive viral status (OR 3.31, 95% CI 2.44, 4.56, p = 2.15x10^-13^). (c) Overall cancer incidence within the SLE cohort is significantly predicted by the viral status (OR 1.76, 95% CI 1.33, 2.31, p = 4.12x10^-4^) and being male (OR 1.65, 95% CI 1.33, 2.03, p = 2.72x10^-5^). (d) Overall cancer incidence within the MS cohort is significantly predicted by viral status (OR 4.65, 95% CI 1.74, 10.41, p = 0.0025).

To uncover the link between autoimmune disorders, viral incidence and cancer incidence, we investigated whether patients with systemic lupus erythematosus (SLE) and multiple sclerosis (MS) had higher incidence of clinically relevant infection with Epstein Barr virus (EBV), Human Papilloma virus (HPV), or Hepatitis B or C (Hep) compared to controls. Only infections that led to a physician visit or laboratory test would be entered into the medical records; most viral infections do not meet these criteria. Positive viral status is therefore likely to be a better proxy for serious infections than for all infections. SLE patients had an increased overall viral incidence (odds ratio (OR) 1.60, 95% CI 1.37, 1.87, p = 1.58 x 10^−6^) than controls. Males (considering both the SLE patients and controls) were more likely to have higher viral incidence (OR 1.58, 95% CI 1.27, 1.95, p = 2.16 x 10^−4^) (Fig 2A). MS patients had an even larger increased viral incidence compared to controls (OR 3.31, 95% CI 2.44, 4.56, p = 2.15 x 10^−13^), however, men within the MS cohort did not demonstrate an increased risk for cancer compared to women (OR 1.10, 95% CI 0.80, 1.49, p >0.99) (Fig 2B). This may be due to a larger number of men included in the MS cohort (n = 1674) compared to the male SLE cohort (n = 255), or because men diagnosed with SLE tend to have severe disease and may be prone to heightened viral incidence. For all logistic regressions, while age was statistically significant, its meaningful significance is unclear as the OR was close to 1.00 for both SLE and MS.

SLE and MS patients have increased viral incidence as well as an autoimmune disorder. Therefore, we examined whether the autoimmune disorder or viral incidence better predicted overall cancer risk by looking at overall cancer risk in both SLE patients and MS patients. SLE patients have increased risk for some cancers and decreased risk for others; therefore it was unsurprising that SLE alone did not significantly predict overall cancer incidence (OR 0.81, 95% CI 0.67, 0.96, p = 0.17) (Fig 2C). Furthermore, the interaction between SLE and overall viral incidence did not predict overall cancer incidence (OR 1.75, 95% CI 1.12, 2.72, p = 0.11) (Fig 2C). However, SLE patients with a positive viral test (OR 1.76, 95% CI 1.33, 2.31, p = 4.12 x 10^−4^) and male SLE patients (OR 1.65, 95% CI 1.33, 2.03, p = 2.72 x 10^−5^) had increased cancer risk (Fig 2C). The interaction between MS and viral status did not predict overall cancer incidence (OR 1.41, 95% CI 0.56, 4.11, p = 1.00) (Fig 2D). While not significant, MS alone showed a trend for increased in risk of overall cancer incidence (OR 1.25, 95% CI 1.05, 1.50, p = 0.059) (Fig 2D). Additionally, gender was not a meaningfully significant contributor for cancer risk in the MS cohort (OR 1.20, 95% CI 0.98, 1.45, p = 0.27) (Fig 2D). As in the SLE patients, viral status significantly predicted overall cancer risk for the MS cohort (OR 4.64, 95% CI 1.74, 10.4, p = 0.0025) (Fig 2D). This suggests that viral status is a more important predictor for cancer development than either autoimmune disorder. Since autoimmune patients have an increased risk for viral incidence, this may explain the heightened risk in SLE patients for virus-associated cancers documented in other studies [6,8,9].

### Hematological cancers

**Fig 3:**
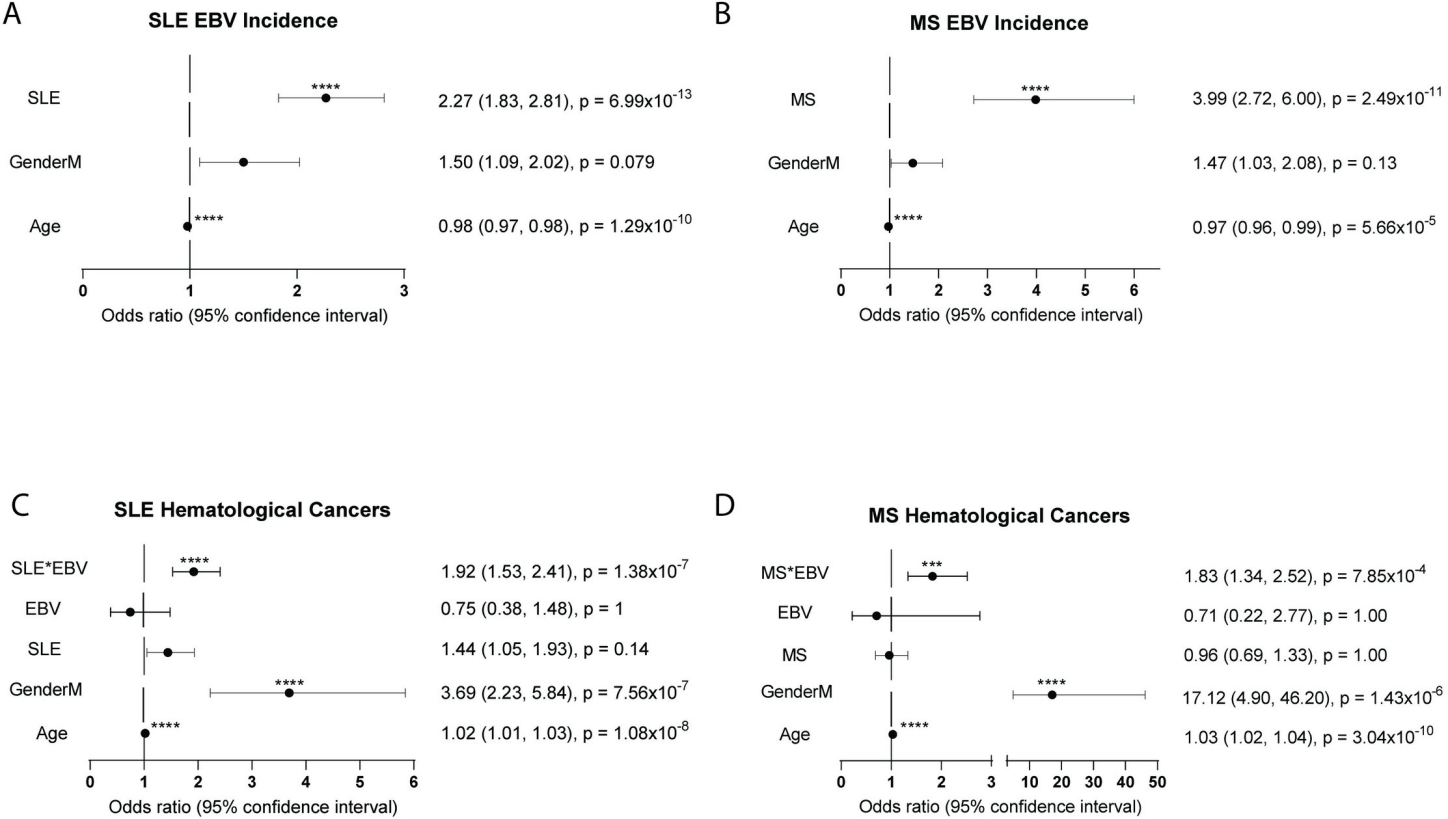
Autoimmune disorders increases risk of EBV infection, and autoimmune disorder with EBV increases risk for hematological cancers. Odds ratios (OR) and 95% confidence intervals of SLE and MS cohorts are shown. For all graphs, the dotted line represents an OR of 1.0. An OR to the right of the dotted line represents an increase in risk. An OR to the left of the solid line represents a reduction in risk. (a) EBV viral incidence for SLE cohort is significantly predicted by SLE (OR 2.27, 95% CI 1.83, 2.81, p = 6.99 x 10^−13^). (b) EBV incidence for MS cohort is significantly predicted by MS (OR 3.99, 95% CI 2.72, 6.00, p = 2.49x10^-11^). (c) Hematological cancer incidence for the SLE cohort is significantly predicted by the interaction of SLE with EBV (OR 1.92, 95% CI 1.53, 2.41, p = 1.28x10^-7^) and male (OR 3.69, 95% CI 2.23, 5.84, p = 7.56x10^-7^). (d) Hematological cancer incidence for the MS cohort is predicted by interaction of MS with EBV (OR 1.83, 95% CI 1.34, 2.52, p = 7.85x10^-4^) and male (OR 17.12, 95% CI 4.90, 46.20, p = 1.43x10^-6^).

To more specifically examine any heightened risk for viral-influenced cancers, we next investigated if viral incidence and SLE or MS increased the risk for hematological cancers. SLE and MS patients were more likely to have tested positive for EBV infection (OR 2.27, 95% CI 1.83, 2.81, p = 6.99 x 10^−13^; and OR 3.99, 95% CI 2.72, 6.00, p = 2.49 x 10^−11^) (Fig 3A and 3B). Male participants in both the SLE and MS cohorts may have a slight, though not significant, increase in EBV incidence (OR 1.50, 95% CI 1.09, 2.02, p = 0.079; and OR 1.47, 95% CI 1.03, 2.08, p = 0.13) (Fig 3A and 3B).

The best predictor for hematological cancer risk was the interaction of SLE or MS with EBV (OR 1.92, 95% CI 1.53, 2.41, p = 1.38 x 10^−7^; and OR 1.83, 95% CI 1.33, 2.52, p = 7.85 x 10^−4^) (Fig 3C and 3D). Men were also significantly more likely to develop hematological cancer in both cohorts (OR 3.69, 95% CI 2.22, 5.84, p = 7.56 x 10^−7^; and OR 17.12, 95% CI 4.90, 46.20, p = 1.43 x 10^−6^) (Fig 3C and 3D). Taken together, these data suggest that patients with SLE or MS are more likely to have clinically relevant EBV infections and these are associated with increased hematological cancer.

We sought to confirm this finding with other SLE-increased cancers including hepatobiliary, anal/vaginal, bladder and thyroid cancers. The MS cohort had surprisingly few tests for these viruses overall (Hep C (n = 29, 0.2%), HPV (n = 43, 0.3%)) compared to the SLE cohort (Hep C (n = 524, 6.5%), HPV (n = 92, 1.1%)) (Methods: Table 1). To compound this problem, the number of patients in both the SLE and MS cohort with cancer and positive viral ICD-9/ICD-10 and lab codes were too low for statistical power to see correlations for these cancers (Methods: Table 1). Therefore, we could not run logistical regression on these cancer and viral combinations.

### Cancers for which SLE and MS patients are at decreased risk

**Fig 4:**
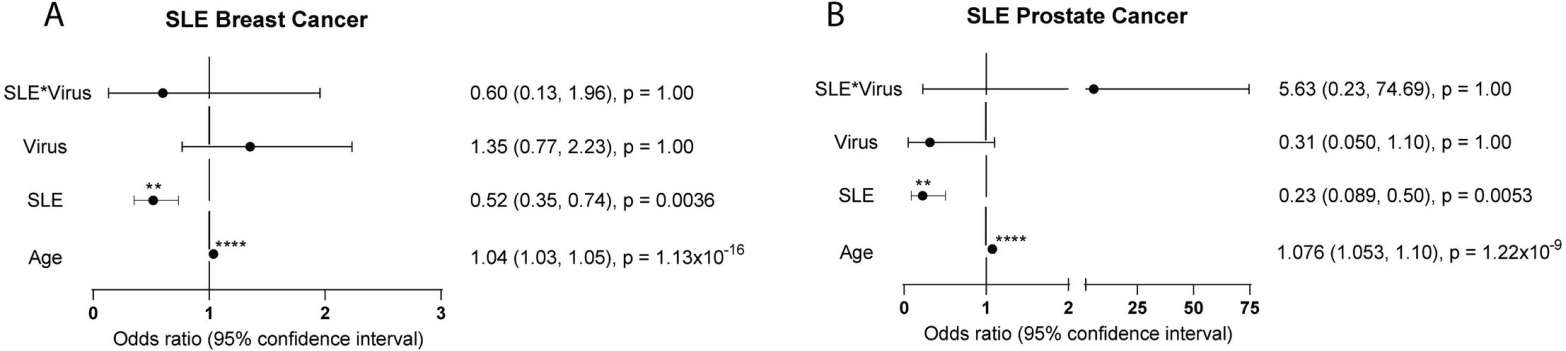
SLE decreases risk for hormonal cancers. Odds ratios (OR) and 95% confidence intervals of SLE and MS cohorts. For all graphs, the dotted line represents an OR of 1.0. An OR to the right of the dotted line represents an increase in risk. An OR to the left of the solid line represents a reduction in risk. (a) Breast cancer incidence in female SLE cohort is significantly decreased by SLE status (OR 0.52, 95% CI 0.35, 0.74, p = 0.0036). (b) Prostate cancer incidence in male SLE cohort is significantly decreased by SLE status (OR 0.23, 95% CI 0.089, 0.50, p = 0.0053).

SLE patients have decreased risk for hormonally-influenced cancers, including breast and prostate cancers. To confirm that our SLE cohort was properly curated and that viruses are not involved in these cancers, we examined how SLE and viral status (HPV, EBV, Hep) affected breast and prostate cancer incidence for women and men, respectively. Breast cancer risk was approximately halved for patients with SLE (OR 0.517, 95% CI 0.35, 0.74, p = 0.0036) (Fig 4A). Neither viral infection alone (OR 1.35, 95% CI 0.77, 2.23, p > 0.99) nor SLE and virus interaction (OR 0.60, 95% CI 0.13, 1.96, p > 0.99) predicted breast cancer incidence. Prostate cancer risk was also greatly reduced for SLE patients (OR 0.22, 95% CI 0.089, 0.50, p = 0.0053) (Fig 4B). Again, neither viruses alone (OR 0.31, 95% CI 0.050, 1.10, p = 0.99) nor SLE and viruses together (OR 5.63, 95% CI 0.23, 74.69, p = 1.00) predicted prostate cancer incidence for the SLE cohort (Fig 4B). In contrast, MS patients had similar breast (n = 89, 1%) and prostate cancer (n = 32, 0.4%) risk compared to control populations (n = 68, 0.9% and n = 37, 0.5% respectively). Logistic regression was not run for MS samples as there was a lack of viral medical records for these patients. This evidence demonstrates that not all cancers are increased in SLE patients and that not all cancer risk is heightened by viral infections.

## Discussion and conclusions

In this study, we found that positive viral infection status is associated with risk for malignancies in general and hematological malignancies in both SLE and MS. SLE and MS patients have a heightened risk of positive viral infection status as noted by laboratory results and ICD-9/10 codes, indicative of increased viral infection incidence or heightened intensity of viral infections compared to controls. SLE patients are at a higher risk than controls for cancers associated with viral infection, but lower or equal risk for other types of malignancies. SLE patients’ overall cancer risk was not significantly different from controls. MS patients showed a different pattern; MS status itself conferred a slightly higher, though not significant, risk for malignancy overall, as well as a higher risk for viral infections. Viral infections continued to have the strongest influence on risk for cancer in the MS patients. Thus, we can conclude that increased susceptibility to more severe viral infection substantially contributes to overall cancer risk in both SLE patients and MS patients.

Systemic lupus erythematosus (SLE) and multiple sclerosis (MS) patients experience increased incidences of some types of cancers including hematological cancers. It is difficult to differentiate whether the disease, medication side effects, or the effects of AD on virus infections are the underlying cause of elevated cancer risk in SLE and MS patients. For example, increased SLE disease activity often leads to intensified drug treatments, making it difficult to tease out the relative contributions of immunosuppressive drugs and SLE itself on cancer risk [6,24]. Cancer diagnosis tends to occur early after SLE diagnosis, often within the first year, suggesting SLE itself influences cancer risk more strongly than drug exposure given the limited time frame for medications to have an effect [25]. Most of the medications used in SLE and MS are immunosuppressive medications. The finding that cancers associated with viral infections are increased in these patients, while those not associated with viral infections are actually decreased in SLE, suggests that the viruses are likely important to causation, even if risk for those viruses is increased as a result of immunosuppressive medication.

The contributions of AD and viral infections to cancer development are particular to the type of cancer. This study shows that SLE and MS patients are more likely to be diagnosed with clinically relevant EBV infection (Fig 2), and hematological cancers are also associated with SLE or MS and EBV infection status. Thus, the risk for hematological cancers may be due to EBV infection, which is associated with Non-Hodgkin’s lymphoma, Burkitt’s lymphoma, and other B cell malignancies [13,14,26]. Control of EBV infection is known to be dysregulated in both SLE and MS, and there is weaker immunity to this virus in these diseases [14,27–33]. Therefore, it is likely that this dysregulation leads to increased risk for hematological cancers. However, as many of the tests used to determine viral status in this study are from serological tests, the timing of viral infection cannot be determined. Further studies to tease apart the temporal relationship between viral infection and malignancy onset are needed.

We confirmed previous findings that suggest that SLE itself confers a lower risk for breast and prostate cancer [34–36]. It is likely that SLE affects hormones or hormone regulation important for the development or proliferation of these cancers [37]. As expected by general population studies, viral infection did not affect the rate of these types of malignancies in the SLE population.

The use of an EHR system allowed access to thousands of records, and made this project possible. However, it also has limitations. Determining what is a “positive viral status” is challenging, and we certainly missed many viral infections. Only infections that led to a physician visit or laboratory test were entered into the medical records; most viral infections do not meet these criteria. Positive viral status is therefore a better proxy for serious infections than for all infections. Some viral infections, such as EBV, may not be commonly noted in the medical records. For example, although nearly all SLE patients are infected with EBV, increased viral replication or viral load, which is common in SLE patients [8,9], would not necessarily be tested for unless it lead to mononucleosis or other symptoms, and would therefore not contribute to a positive viral status for this work. This may have resulted in an underestimation of EBV cases in both SLE and healthy patients and may have affected the results regarding hematological cancers and HPV-associated cancers. Furthermore, SLE and MS patients are commonly screened for hepatitis B/C before beginning immunosuppressive medications, therefore these viruses may have been oversampled compared to the control population. As we relied on clinical notes from the EHR, and disease activity measures are not routinely collected in clinical practice, we could not assess the effect of disease activity on risk for viral infection or malignancy.

Finally, due to the success of early screening programs in the United States, cervical cancer is relatively rare, as are the other HPV-associated cancers. Instead of cervical cancer, therefore, we used cervical changes as an indicator for cervical malignancy. This likely altered the final HPV incidences since this cancer indicator would have been used to denote positive viral status, and HPV infections that do not lead to abnormalities are less likely to be noted.

Our findings indicate that increased viral infection in SLE and MS patients better explains the elevated risk of certain cancers than SLE and MS disease alone. However, patients with MS had a slight increased risk of malignancies separate from viral risk factors, highlighting that different ADs may uniquely affect cancer risks. Both SLE and MS patients had increased risk for hematologic cancers, which were also affected by risk for viral infection. The increased rate of viral infection seems to overcome a general decrease in risk for cancers in SLE patients, leading to an equivalent overall malignancy risk compared to controls. Therefore, special care should be taken with viral infections in SLE patients. Although treatment for EBV infection is currently limited to monitoring patients with active infections, there are vaccines for hepatitis B and HPV and treatment for hepatitis C. It is likely that these vaccines and treatments would especially benefit SLE patients by reducing the associated risk for malignancy.
